# Quality Assessment and Modulating Factors on Self-Regulatory Behavior in Peer-Assisted Medical Learning

**DOI:** 10.3390/healthcare11152223

**Published:** 2023-08-07

**Authors:** Jannis Achenbach, Laura Nockelmann, Michaela Thomas, Thorsten Schäfer

**Affiliations:** Center for Medical Education, Ruhr-University Bochum, 44801 Bochum, Germany; lauranockelmann@t-online.de (L.N.); michaela.thomas@rub.de (M.T.); thorsten.schaefer@rub.de (T.S.)

**Keywords:** quality assessment, peer-assisted learning, skills labs, self-assessment

## Abstract

Objectives: Standardized extracurricular skills labs courses have been developed in recent decades and are important approaches in peer-assisted medical learning (PAL). To provide high quality training and achieve effective learning strategies, continuous evaluations and quality assessments are essential. This research aims to evaluate quality data from medical students participating in extracurricular skills labs courses at Ruhr-University Bochum to prospectively optimize concepts and didactical training and standardize processes. Additionally, we set out to assess and quantify drivers that are influencing factors of the self-reflection of competencies. Methods: The analysis was based on a routine assessment of *n* = 503 attendees of the PAL courses in the skills labs in three consecutive semesters, who voluntarily participated in the evaluation. We analyzed the effects of age, semester and their interaction on the self-reflection of competencies in technical skills courses using moderated regression and simple slope analyses, as previously published. A univariate analysis of variance (ANOVA) with post hoc Tukey HSD testing was used to analyze group means in estimated competencies using *IBM SPSS Statistics V.28*. Results: An analysis of variance revealed a significant increase in self-assessed competencies when comparing *pre*- vs. *post*-course evaluation data in all 35 depicted items *(all p < 0.001).* A total of 65.5% of the items were adjusted significantly differently, revealing modified self-reflected pre-course levels compared to those stated before. A moderated regression analysis revealed that age *(R^2^ = 0.001, F(1;2347) = 1.88, p < 0.665)*, semester of study *(∆R^2^ = 0.001*, *∆F (1;2346) = 0.012, p < 0.912)* and their interaction *(∆R^2^ = 0.001*, *∆F (1;2345) = 10.72*, *p < 0.227)* did not explain a significant amount of the variance in self-reflection variance. A simple slope analysis of earlier *(b = 0.07*, *t = 0.29*, *p < 0.773)* and later semesters of study *(b = 0.06*, *t = 0.07*, *p < 0.941)* did not differentiate from zero. Conclusions: The presented evaluation paradigm proved to be a useful tool to encourage students to initiate self-regulatory and self-reflective behavior. The cohesive evaluation of the large cohort of attendees in extracurricular, facultative skills labs courses was helpful in terms of quality assessments and future adaptations. Further evaluation paradigms should be implemented to assess other influencing factors, such as gender, on self-reflection, since age and semester did not explain significant differences in the model.

## 1. Introduction

### 1.1. Peer-Assisted Learning in the Context of Medical Education

Peer-assisted learning (PAL) is defined as a teaching method whereby students (“peers”) replace the professional educator, helping teach other students specific skills and to assist them by achieving competencies [1,2]. Especially in medical education, PAL is a frequently used method in curricular as well as extracurricular training at medical faculties [3]. Hereby, the effectiveness of the method was evaluated and PAL was depicted as an important and sufficient approach in medical education [1,4,5]. Although many research approaches have been suggested, earlier investigations postulated to extend knowledge on influencing factors of PAL and evaluate concepts with regard to the different participating actors and perspectives [3].

### 1.2. The National Competence-Based Catalogue of Learning Objectives for Medicine (NKLM 2.0)

The guidelines of the National Competence-based Catalogue of Learning Objectives for Medicine (NKLM 2.0) of the German Medical Faculty Association (MFT) highlight competence-based practical medical education (CBME) as the main and most important approach [6,7,8]. To implement competency-based educational concepts based on recommendations in the NKLM, didactic strategies have evolved aiming to generate improved practical teaching competencies [9]. Hereby, investigations have focused on the identification of practical learning goals to standardize the teaching methods of practical approaches in medical faculties [10]. In this context and for the training of clinical competencies in a riskless situation prior to the application in patients, skills laboratories (skills labs) have been developed as equipped practical rooms providing medical students and teachers adequate learning situations and a beneficial atmosphere to improve their competencies [11].

### 1.3. Training in Skills Labs

In Germany, most medical faculties (>90%) have established skills labs in recent decades. However, a need for improving methods and studies in terms of standardization, quality assessments and developing teaching concepts has arisen regarding PAL concepts in the skills labs landscape [12]. These needs have not only been raised by medical faculties and educators but also in nursing and other health professional educational contexts [13,14].

Although many concepts have been developed in the past in terms of evaluated self-regulated and competency-based learning among participating students [15,16,17,18,19,20] and research has focused on the development and implementation of PAL approaches as a common didactical method, an urgent need to standardize concepts, didactical methods and professional teachers as well as content training has been described [12,21,22,23].

A focus has been placed on the assessment of evaluation concepts and data coming from participants in practical, peer-assisted, extracurricular training in skills courses [2,24]. To evaluate reflected competencies, recently, approaches have focused not only on the self-assessment of competencies before and after course participation, but additionally on the imaginary (*self-reflected*) view prior to course participation [20,25]. Hereby, an appropriate self-reflection of competences—as a non-technical and metacognitive skill—is not only important in terms of patient safety and the learning process, but additionally because it is named as part of the NKLM, so that, as other researchers postulated, medical students can be longitudinally trained [26,27].

We set out to evaluate quality assessment data from medical students participating in extracurricular skills labs courses at the Ruhr-University Bochum. With this, we aimed to identify certain components which might prospectively optimize concepts and didactical training as well as standardize processes. This might be transferable to other medical faculties. This research quantifies influencing factors of self-assessed properties with regard to self-regulatory behavior of practical competencies. We evaluated technical concepts to identify course-specific aspects, since earlier interventions analyzed the self-regulatory behavior of participants in the first aid and physical examination course [20,28].

## 2. Methods

### 2.1. Evaluation Data from Peer-Assisted Skills Labs Courses

We investigated data from *n* = 503 attendees of PAL courses at the Ruhr-University Bochum. We analyzed data from three semesters between 2018 and 2020 from students voluntarily participating in the evaluation of our skills labs. Participants were from all different semesters of the entire medical program of the faculty. As a first approach, we analyzed the demographic data of participants.

Standardized peer-assisted courses were exclusively delivered by peers after they had been trained by didactical and medical experts in the field of the specific course content. Therefore, structured course manuals were implemented.

#### The Evaluation Paradigm

The evaluation concept consisted of a self-assessment of competencies before (1: *pre*), after (2: *post*), and after the course with a retrospective assessment of competencies before participating in the course (3: *post-pre*) as previously published [20,25]. Next to group comparisons of *pre*- vs. *post*-, *pre*- vs. *post-pre and post*- vs. *post-pre-evaluation* data, we analyzed the influence of age, semester of study and its interaction on the self-reflection of competencies, depicted as the concordant self-reflection as earlier described [20]. The latter was defined as the difference of the *post-pre*- minus *pre*- assessment. Negative scores indicate that participants reveled a more negative (critical) assessment of their own competencies before the course and afterwards retrospectively adjusted these scores to better self-assessed competencies [20]. Higher positive data revealed that participants evaluated themselves as having better self-assessed competencies before the course than retrospectively. Here, we assessed evaluations from technical skills courses (*n* = 335 participants), implementing concepts for teaching blood collection, surgery, peripheral indwelling catheters, changing bandages and central indwelling catheters.

Evaluation included, in each course, a survey of different course-specific items and a self-assessment, documented using a six-point Likert scale differentiating between strongly agree = 1 and strongly disagree = 6 as the most severe expressions, as previously published [20,28]. Students were asked if they felt confident in dealing with the respective specific competence; lower numbers (1) meant better competencies and higher numbers (6) revealed lower competencies. The overall evaluation was also assessed by a six-point Likert scale; however, it was oriented with a score of 1 being very good and 6 being very poor. Data were evaluated as part of the routine course assessments.

Pseudonymized evaluation data were assessed routinely as part of the quality and evaluation assessments and collection and analysis were conducted anonymously.

### 2.2. Statistical Analysis

We implemented a statistical approach, as previously published and described in our earlier work. Group means and standard deviation were evaluated using a univariate analysis of variance (ANOVA) with post hoc Tukey HSD testing for pairwise analysis of different estimates on the self-assessment of competencies in IBM SPSS Statistics V.28. Adjustment for multiple testing was applied using Bonferroni corrections. Dependent variables were tested for normal distribution using the Kolmogorov–Smirnov test. The homogeneity of variances was asserted using Levene’s Test. For unequal variances, values were reported with Welch’s test. Chi-square tests were used for analyses of categorical and descriptive variables. A moderated regression analysis was conducted to analyze the interaction effect of age and semester of study, as well as their interaction, on the concordant self-reflection of competencies in technical course concepts. Simple slope analyses were calculated to illustrate the effects [20].

## 3. Results

### 3.1. Demographic Data of Participants in Peer-Assisted Extracurricular Course Concepts

The following table provides participant ages, semesters of study and whether participants were professionally trained previously or had participated in the courses before (Table 1).

Regarding different course domains, most participants attended one of the technical courses (66.4% of all participants). In total, *n* = 503 students participated in courses and the evaluation (Table 2).

**Figure 1:**
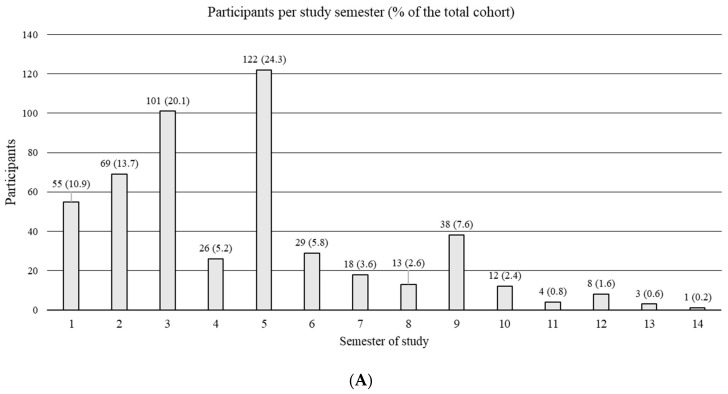

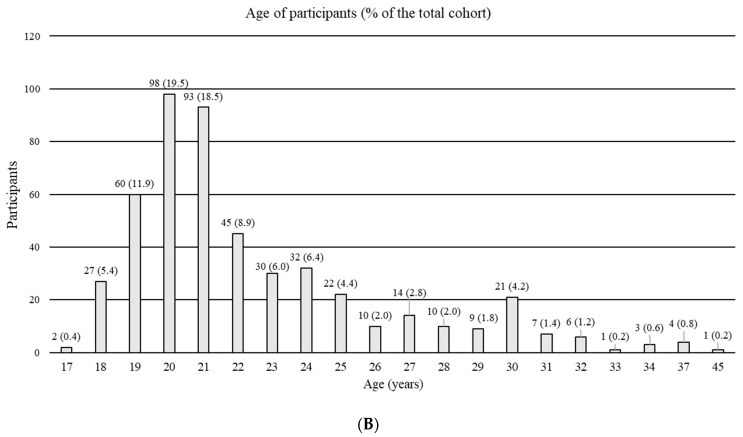
Distribution of participants: semester affiliation (**A**) and age (**B**).

An analysis of participants with regard to the study semester revealed that most participants attended during their fifth semester (24.3% of all participants) and the majority of all participants were 20 (19.5% of all participants) or 21 (18.5% of all participants) years old (Figure 1).

### 3.2. Evaluation Regarding Quality Assessment Data

As a first approach, we assessed data from the general and non-course-specific part of the presented evaluation paradigm, documenting attendees’ satisfaction and overall impression with regard to their course participation. Data were surveyed in ten items, as well as two concluding questions, with regard to the overall course evaluation (the results are documented in Table 3).

### 3.3. Analysis of Self-Reflected Competencies in Technical Course Concepts

Following this, we assessed evaluation data from *N* = 335 participants in the technical course concepts with regard to attendees *pre*-, *post*- and *post-pre*-evaluation. In all 35 out of 35 assessed domains from the five course concepts, participants revealed a significant increase in self-assessed competencies with regard to *pre*- vs. *post*-evaluation data (all *p < 0.001).* The same differences were depicted for the *post-pre*- vs. *post*-data (all *p < 0.005*). Regarding the *pre*- vs. *post-pre*-evaluation, a significant change in the self-assessment was observed in 23 out of 35 domains (Table 4).

### 3.4. Analysis of Significantly Different Pre- vs. Post-Pre-Comparisons

**Figure 2:**
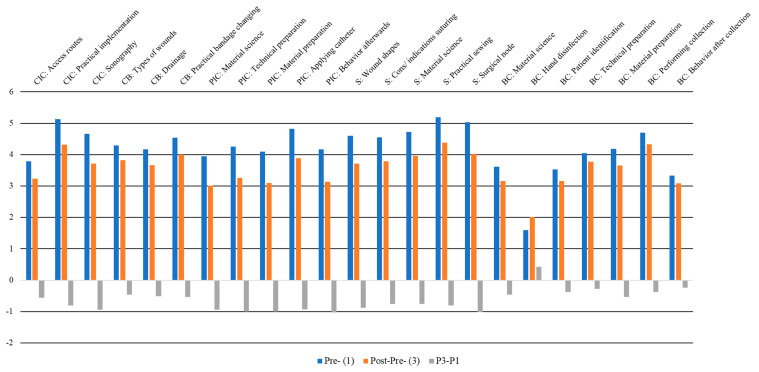
Comparison of differentiating *pre*- (blue) vs. *post-pre* (orange) evaluations depicted for statistically differentiating domains. Differences in *post-pre* minus *pre*-data are presented in gray (negative scores = a more critically assessment before the course, corrected afterwards to better pre-levels). *Y*-axis: Points and differences on the six-point Likert scale; *X*-axis: Different course concepts. Abbreviations: CIC, central indwelling catheter; CB, changing bandages; PIC, peripheral indwelling catheter; S, surgery; BC, blood collection.

The comparison of *pre*- vs. *post-pre*-data was assessed as self-regulatory behavior. Out of 35 domains, students significantly evaluated *post-pre*-levels differently in 23 domains, revealing a retrospectively adjusted pre-competency level. Remarkably, in 22 of these items, attendees retrospectively assessed their pre-competencies as better than before. In the following, all significantly differentiating *pre*- vs. *post-pre*-items are depicted. The differences in *post-pre* minus *pre*-data were analyzed, revealing negative scores as a more critical assessment before the course, which were afterwards corrected to improved pre-levels (Figure 2).

### 3.5. Moderating Effects of Age, Semester of Study and Their Interaction on the Self-Regulatory Behavior of Competencies

**Figure 3:**
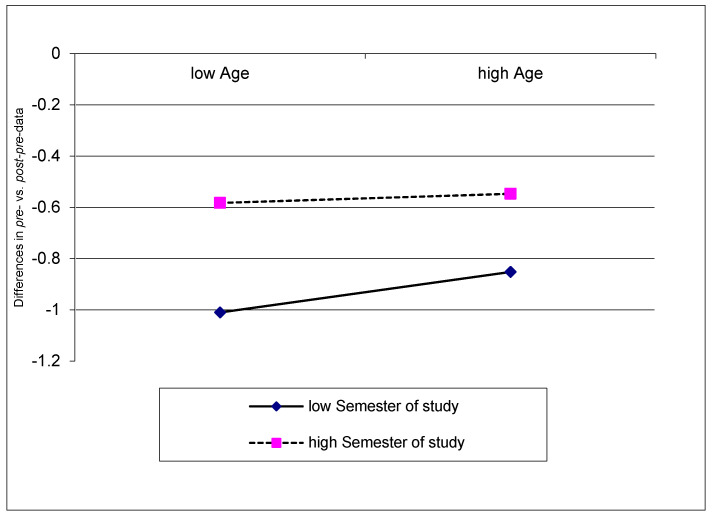
Analysis of interaction effects of age and semester of study on the self-regulatory assessments in technical courses illustrated as simple slopes (0 = no differences between *pre*- and *post-pre*-evaluations; *negative scores* = a more critically *pre*-assessment, retrospectively adjusted to better *pre*-competencies).

As a further approach, we set out to analyze the effects of age, study semester and their interaction on self-regulatory behavior using a moderated regression analysis. To analyze the self-regulatory behavior depicted as the difference in *post-pre*- minus *pre*-assessment, we assessed data from all five assessed technical courses, revealing an overall number of *2349* reflected items. The moderated regression analysis revealed that age (*R^2^* = 0.001, *F*(1;2347) = 1.88, *p* < 0.665), semester of study (*∆R^2^* = 0.001, *∆F* (1;2346) = 0.012, *p* < 0.912) and the interaction of both (*∆R^2^* = 0.001, *∆F* (1;2345) = 10.72, *p* < 0.227) did not explain a significant amount of variance in the self-reflected variance (*F* = 0.55, *p* < 0.646). A simple slope analysis was conducted to illustrate the effects in a figure. Slopes for a tendentially low semester of study (*b* = 0.07, *t* = 0.29, *p* < 0.773) and high semester (*b* = 0.06, *t* = 0.07, *p* < 0.941) did not significantly differentiate from zero. Illustrations revealed that participants tended to have a more critical assessment of their competencies before the course and this was afterwards retrospectively adjusted to better self-assessed pre-competencies. This was, however, independent from the age and semester of participants (Figure 3).

## 4. Discussion

Competency-based and practical medical learning are important approaches in current medical education paradigms that require standardized concepts and a longitudinal evaluation in terms of continuous quality assessments and objectifiable principles [29,30]. The presented investigation aimed to evaluate quality data from course attendees from a large student cohort participating in extracurricular PAL at the Ruhr-University Bochum to objectify the current satisfaction of participants. Furthermore, we aimed to objectify the influencing factors of self-regulatory learning and depict course-specific factors. Hereby, the self-regulatory behavior and self-reflection of skills is known as a metacognitive process and is an essential tool in adequate learning processes and successful medical learning [27]. A better understanding of influencing factors on self-assessment and self-reflection was previously determined as fundamental for effective practical skill training; however, research has determined multiple influencing and partly unknown factors that may play a bias or modulating role [31].

As a first result, we identified by an analysis of demographic attendees’ data that participants of the extracurricular courses predominantly attended in their first five semesters of study (about 74% of all participants), whereby more than one-third (38%) was 20 or 21 years old. With regard to earlier investigations, which described that age is associated with a better developed personality and trained emotional regulative processes [32], this could conversely mean that students at a comparatively younger age are still in the developmental and learning process, meaning practical teaching methods may positively influence older health professionals. Thereafter, the course takes place at an appropriate time. At the same time, the findings suggest that further concepts for students in later semesters, implementing, e.g., more complex skills, should be offered to expand the training courses in later semesters or to older students. The course concept regarding the central venous catheter insertion—as published earlier—is a more complex course which might be a suitable approach and should further be expanded. [16,33] A further assessment of the overall quality evaluation data revealed that participants were satisfied after participating in the courses (99.8% recommended the course), which is explainable by the standardized concepts and the earlier continuous evaluation strategy. Standardized concepts can be used as a useful starting point to integrate innovative learning methods or technical adaptations in future.

The main focus was set on the evaluation of self-assessed competencies, whereby the evaluation concept proved that participants subjectively benefitted from course participation. This was documented in all assessed 35 evaluation items.

### Limitations

However, as a limitation of the presented data here, no objectifiable measures of competencies are given; thus, future approaches should expand the focus to practical competency increases, e.g., by assessing the skills of participants and discrepancies between the self-perceived competencies. An appropriate approach could be to evaluate competencies in summative tests like “Objective Structured Clinical Examinations (OSCE)” which is currently being considered for implementation in curricular medical trainings with regard to the implementation of new licensing regulations for medical professionals. This limitation mainly concerns the assessment of trained competencies, whereby an assessment of self-regulatory behavior itself seems difficult to evaluate. 

As a main advantage of the presented data, not only were *pre*- vs. *post*-assessments given, but also *self-reflected pre*-competencies. Hereby, about 65.5% of the assessments significantly differed when compared to the *pre*-data. This is nearly congruent with previous assessments of first aid and examination courses, whereby 72.7% differed significantly. [20] The fact that more than two-thirds of the participants retrospectively adjusted their *pre*-competencies suggests that the evaluation paradigm forced students to make use of self-reflective or self-regulated behavior. Since the ability for self-reflection was previously postulated as an important value for older health professionals, the evaluation concept should additionally be expanded in the courses as a useful tool to train medical students in self-regulatory competencies [34].

Remarkably, in nearly all assessments (22 out of 23 items), students corrected their initial *pre*-levels retrospectively to better *pre*-course levels (depicted in the *post-pre* evaluation). This might be explainable at least partly by gender. An analysis of demographic data revealed that more than two-thirds (67.9%) of the attendees were female. Earlier research postulated that gender has an influence on self-regulatory behavior. [35,36,37] Hereafter, it might be conceivable that the majority of female students initially had a more critical self-assessment of competencies and noticed after participating in the course that the earlier assessed competency was underestimated with regard to the specific item. Future research should therefore focus additionally on gender-specific aspects to extend the knowledge of influencing factors. Notably, no significant effects were observed based on the influence of age, semester of study and their interaction on the reflected self-assessment, which is contrary to earlier investigations of other concepts [20]. It is reasonable that course-specific, gender-specific and other undetected aspects may have had an influence, and overlapping effects of age, semester and their interaction were amended. In particular, the technical course format consists of shorter units, whereby examinations and first aid courses are held in different timed modules on different days. This might have had an influence as well.

As a further limitation, other—and to date unknown—factors may be involved which have not been further assessed, since self-reflection was evaluated as part of the established routine evaluation design and no further specific questionnaires were implemented. Specific scales have previously been described for the assessment of self-reflection competencies, such as the “Self-Regulation of Learning Self-Report Scale (SRL-SRS)”, and should therefore be implemented in future assessments. [38]

The depicted illustrated graphs in the simple slope analysis proved to be a useful psychometric tool to document that participants tended to have a more critical assessment of their own competencies before course participation, which afterwards adjusted to better pre-course competencies, regardless of age and semester of study, which is therewith partly contrary to earlier findings [20].

## 5. Conclusions

The presented evaluation paradigm is a useful tool to encourage students to initiate self-regulatory and self-reflective behavior. The first cohesive evaluation of the presented large cohort of attendees in our extracurricular, facultative skills labs was helpful in terms of a quality assessment and future adaptations. Further longitudinal evaluation paradigms will be implemented with standardized questionnaires for an assessment of self-regulatory skills and should objectify trained practical skills. Other influencing factors of self-reflection of skills in medical education, such as gender, should be analyzed, since age and semester did not explain significant differences in the analyzed model.

## Figures and Tables

**Table 1:** Descriptive statistics of attendees’ demographics.

Domain/Variable	*M*	*SD*	*Range*
Age (y) (*n* = 495)	22.46	3.91	17–45
Semester of study (*n* = 499)	4.55	2.75	1–14
Sex (*n* = 502)	***Female*** 341	***Male*** 161	***% Female*** 67.9
Earlier professional training (*n* = 481)	***Yes*** 83	***No*** 398	***% Yes*** 17.3
Earlier participation in skills labs course (*n* = 490)	335	155	68.4

**Table 2:** Peer-assisted course concepts and the number of participants in the three consecutive semesters.

Course Domain	Peer-Assisted Course Concept	*Participants*	*%*
Technical courses	Blood collection	100	19.9
	Surgery	58	11.5
	Peripheral indwelling catheter	88	17.5
	Changing bandages	35	7.0
	Central indwelling catheter	53	10.5
		*Σ 334*	*Σ 66.4*
Emergency courses	First aid	129	25.6
Clinical assessment courses	Examination course	40	8.0

**Table 3:** Overall evaluation of satisfaction with regard to different domains from course participants.

Domain/Variable	*M*	*SD*	*Range*
Working atmosphere (*n* = 492)	1.08	0.27	1–3
Organizational framework	1.27	0.53	1–4
Course structure (clear and understandable)	1.19	0.41	1–3
Preparation for the professional practice	1.55	0.70	1–5
Targeted and specific work orders	1.21	0.43	1–3
Use of technical aids in the tutorial	1.36	0.69	1–6
Possibilities for own practical work	1.11	0.33	1–3
Integration of theoretical parts	1.29	0.52	1–4
Peer tutors regarding methodical-didactic competencies	1.18	0.40	1–3
Peer tutors regarding role model function	1.16	0.40	1–3
Would you recommend the course?	***Yes*** 491	***No*** 1	***% Yes*** 99.8
How is your overall course evaluation?	***Great*** 487	***Quite okay*** 5	***Changes needed*** 0

**Table 4:** Analysis of self-reflection of competencies with regard to course-specific domains (*Pre*-, *Post*-, and *Post-Pre*-Scores: 1 = very good to 6 = very poor).

Domain/Item	Pre- (1)	Post (2)	Post-Pre- (3)	*p* (1) vs. *p* (2)			*p* (1) vs. *p* (3)			*p* (2) vs. *p* (3)		
Blood Collection (*N* = 100)				*p*	*F*	*Part. Eta2*	*p*	*F*	*Part. Eta2*	*p*	*F*	*Part. Eta2*
Material science	3.62 (1.42)	1.44 (0.57)	3.16 (1.48)	**<0.001**	264.64	0.714	**<0.001**	17.63	0.151	**<0.001**	154.02	0.609
Hand disinfection	1.60 (0.67)	1.20 (0.45)	2.02 (1.07)	**<0.001**	31.68	0.242	**<0.001**	18.12	0.155	**<0.001**	67.40	0.405
Patient identification	3.53 (1.51)	1.48 (0.73)	3.16 (1.69)	**<0.001**	175.73	0.640	**0.011**	6.73	0.064	**<0.001**	116.54	0.541
Protective measures	2.98 (1.36)	1.32 (0.57)	2.86 (1.38)	**<0.001**	149.30	0.604	**0.330**	0.969	0.010	**<0.001**	131.00	0.570
Technical preparation	4.05 (1.44)	1.61 (0.69)	3.77 (1.65)	**<0.001**	302.82	0.754	**0.022**	5.38	0.052	**<0.001**	178.03	0.643
Material preparation	4.18 (1.66)	1.34 (0.55)	3.65 (1.59)	**<0.001**	322.70	0.765	**<0.001**	14.57	0.128	**<0.001**	224.42	0.694
Performing collection	4.70 (1.45)	2.07 (0.79)	4.33 (1.55)	**<0.001**	288.55	0.745	**<0.005**	10.99	0.100	**<0.001**	209.60	0.679
Behavior after collection	3.33 (1.48)	1.29 (0.52)	3.09 (1.48)	**<0.001**	202.12	0.671	**0.040**	4.31	0.042	**<0.001**	158.79	0.616
**Surgery (*N* = 58)**												
Wound shapes	4.60 (1.28)	2.29 (0.88)	3.72 (1.35)	**<0.001**	175.74	0.755	**<0.001**	23.20	0.289	**<0.001**	61.42	0.519
Cons/indications suturing	4.55 (1.14)	2.27 (0.72)	3.79 (1.35)	**<0.001**	209.88	0.786	**<0.001**	21.96	0.278	**<0.001**	72.84	0.561
Material science	4.72 (1.18)	1.88 (0.90)	3.96 (1.54)	**<0.001**	258.25	0.819	**<0.001**	15.27	0.211	**<0.001**	86.38	0.602
Practical sewing	5.19 (1.11)	2.21 (0.81)	4.38 (1.58)	**<0.001**	382.07	0.870	**<0.001**	14.58	0.204	**<0.001**	94.98	0.625
Surgical node	5.03 (1.24)	1.81 (0.71)	4.02 (1.75)	**<0.001**	330.17	0.853	**<0.001**	19.33	0.253	**<0.001**	82.35	0.591
Sterile washing	3.90 (1.56)	1.77 (0.73)	3.65 (1.65)	**<0.001**	117.86	0.674	**0.215**	1.57	0.027	**<0.001**	74.77	0.567
Hygiene measures	3.48 (1.66)	1.84 (0.74)	3.43 (1.80)	**<0.001**	68.54	0.564	**0.742**	0.109	0.002	**<0.001**	58.55	0.507
**Peripheral indwelling catheter (*N* = 88)**												
Material science	3.95 (1.58)	1.64 (0.75)	3.01 (1.76)	**<0.001**	217.58	0.714	**<0.001**	29.12	0.254	**<0.001**	60.71	0.414
Hand disinfection	1.59 (0.87)	1.26 (0.51)	1.53 (0.85)	**<0.001**	15.00	0.147	**0.539**	0.380	0.004	**<0.005**	9.59	0.100
Protective measures	2.28 (1.19)	1.39 (0.67)	1.98 (1.15)	**<0.001**	50.60	0.368	**0.062**	3.59	0.040	**<0.001**	24.46	0.221
Technical preparation	4.26 (1.52)	1.79 (0.86)	3.26 (1.69)	**<0.001**	267.71	0.755	**<0.001**	31.17	0.266	**<0.001**	70.51	0.451
Material preparation	4.10 (1.61)	1.45 (0.74)	3.10 (1.83)	**<0.001**	249.21	0.743	**<0.001**	27.15	0.242	**<0.001**	81.45	0.486
Applying catheter	4.82 (1.43)	2.44 (1.08)	3.89 (1.77)	**<0.001**	215.57	0.715	**<0.001**	27.48	0.244	**<0.001**	86.46	0.501
Behavior afterwards	4.17 (1.65)	1.68 (0.89)	3.14 (1.73)	**<0.001**	209.82	0.707	**<0.001**	34.96	0.289	**<0.001**	77.24	0.473
**Changing bandages (*N* = 35)**												
Material science	3.97 (1.33)	1.97 (0.85)	3.60 (1.44)	**<0.001**	99.17	0.745	**0.074**	3.41	0.091	**<0.001**	49.18	5.91
Hygiene measures	3.63 (1.39)	1.57 (0.70)	3.57 (1.33)	**<0.001**	78.83	0.699	**0.763**	0.093	0.003	**<0.001**	76.77	0.693
Types of wounds	4.29 (1.29)	2.70 (1.14)	3.83 (1.42)	**<0.001**	52.18	0.613	**0.023**	5.67	0.143	**<0.001**	28.25	0.461
Cardinal signs	3.14 (1.42)	1.77 (0.69)	2.77 (1.48)	**<0.001**	30.18	0.470	**0.091**	3.03	0.082	**<0.001**	15.66	0.315
Drainage	4.17 (1.40)	2.17 (0.89)	3.66 (1.45)	**<0.001**	61.03	0.642	**0.037**	4.71	0.122	**<0.001**	34.23	0.502
Practical bandage changing	4.54 (1.17)	1.97 (0.78)	4.00 (1.63)	**<0.001**	129.90	0.793	**0.016**	6.41	0.159	**<0.001**	50.50	0.598
**Central indwelling catheter (*N* = 54)**												
Surgical node	2.90 (1.35)	2.11 (1.18)	3.07 (1.53)	**<0.001**	48.95	0.480	**0.111**	2.62	0.047	**<0.001**	63.21	0.544
Access routes	3.79 (1.40)	1.83 (0.69)	3.23 (1.27)	**<0.001**	100.25	0.654	**<0.005**	12.22	0.187	**<0.001**	69.15	0.566
Cons/indications	3.74 (1.28)	1.73 (0.62)	3.43 (1.47)	**<0.001**	184.64	0.777	**0.064**	3.56	0.063	**<0.001**	81.57	0.606
Alternative venous accesses	3.11 (1.31)	1.49 (0.57)	3.15 (1.51)	**<0.001**	102.21	0.656	**0.830**	0.046	0.001	**<0.001**	74.51	0.584
Theoretical procedure	3.92 (1.37)	1.55 (0.63)	3.73 (1.63)	**<0.001**	182.87	0.775	**0.317**	1.02	0.019	**<0.001**	87.10	0.626
Practical implementation	5.13 (1.15)	1.87 (0.23)	4.32 (1.60)	**<0.001**	410.40	0.886	**<0.001**	20.45	0.278	**<0.001**	123.56	0.700
Sonography	4.66 (1.50)	1.92 (0.80)	3.72 (1.67)	**<0.001**	248.22	0.824	**<0.001**	23.40	0.306	**<0.001**	74.93	0.586

## Data Availability

The data that support the findings of this study are available from the corresponding author upon reasonable request. The data are not publicly available.
